# Ventriculoperitoneal shunt distal dysfunction due to peritoneal malabsorption: etiologies and management strategies—An institutional review

**DOI:** 10.1007/s10143-026-04176-2

**Published:** 2026-02-23

**Authors:** Muhammad Ateya, Mehmet Saim Kazan, Ahmet Özak, Zafer Erdoğan

**Affiliations:** Department Of Neurosurgery, Akdeniz University Hospital, Antalya, Türkiye

**Keywords:** Ventriculoperitoneal shunt, Pseudocyst, Non-hepatic ascites, Axial deformity, Revision surgery

## Abstract

This study aims to investigate the etiology, risk factors, and management outcomes of peritoneal malabsorption, including pseudocysts and non-hepatic ascites, as a complication of ventriculoperitoneal (VP) shunting in pediatric hydrocephalus patients, with a focus on identifying predictors of distal dysfunction and evaluating therapeutic strategies. A retrospective review was conducted on 81 unique pediatric patients (≤ 18 years) undergoing 113 VP shunt revision episodes at Akdeniz University Hospital (2019–2023). Variables included age, sex, catheter site, hydrocephalus type, revision history, axial deformity severity, and abdominal surgery history. Peritoneal malabsorption occurred in 10 patients (11 revision episodes; 10 pseudocysts and 1 non-hepatic ascites), were analyzed for risk factors and management outcomes, with a minimum 9-month follow-up using ultrasonography and CT scans. Peritoneal malabsorption occurred in 10 patients (11 revision episodes), with statistically significant risk factors including history of multiple previous revisions (OR 8.02, 95% CI 1.86–34.63, *p* = 0.002), prior peritoneal-breaching abdominal surgery (OR 11.81, 95% CI 2.73–51.04, *p* = 0.001), and axial deformity (OR 4.07, 95% CI 1.03–16.03, *p* = 0.034; 2.77-fold increase per severity grade, *p* = 0.027). Prior abdominal surgery was present in 60% of malabsorption patients vs. 11.3% without, including even minor procedures (e.g., PEG placement, appendectomy, inguinal hernia repair). Management achieved peritoneal salvage in 7 of 10 patients; 5 episodes required only one procedure, while 6 episodes involved multiple interventions, with 3 ultimately needing ventriculoatrial shunt conversion. This small single-center series identifies prior abdominal surgery, axial deformity, and history of multiple previous revisions as factors associated with peritoneal malabsorption in VP shunts, suggesting mechanical and inflammatory contributions. Management remains challenging, with variable success rates and significantly prolonged hospitalization. These findings highlight the need for preoperative risk assessment and larger studies to refine therapeutic strategies.

## Introduction 

Cerebrospinal fluid (CSF) is a clear ultrafiltrate of plasma that circulates within the ventricles and subarachnoid spaces, providing mechanical protection and contributing to central nervous system homeostasis [1]. In adults, the total volume is approximately 150 mL—about 125 mL in the subarachnoid compartment and 25 mL in the ventricles—renewed four to five times daily [2]. In children, total CSF volume is lower and age-dependent, typically ranging from 60 to 100 mL in older children (approaching adult values by adolescence), with estimates of 65–150 mL in ages 4–13 years and substantially less in younger children and infants [3]. Most CSF is produced by the choroid plexus, with smaller extrachoroidal contributions [4], through active ion transport involving carbonic anhydrase. From the lateral ventricles, CSF flows to the third and fourth ventricles, propelled by ependymal cilia [5], cardiac and respiratory pulsations [6], before exiting via the foramina of Magendie and Luschka into the spinal and cortical subarachnoid spaces. Reabsorption occurs primarily through arachnoid granulations into the venous sinuses [7]. When CSF dynamics are disrupted, as in hydrocephalus, diversion becomes essential. In ventriculoperitoneal (VP) shunting, CSF is directed to the peritoneal cavity, where absorption mimics peritoneal dialysis and relies on the integrity of the mesothelial lining. Chronic exposure to inflammatory stimuli, fibrosis, and repeated surgical interventions—as seen in peritoneal dialysis patients—can lead to mesothelial denudation, basement membrane loss, and vasculopathy [8]. These structural changes impair absorptive capacity and may contribute to shunt failure, pseudocyst formation, or the need to abandon the peritoneal cavity as a distal site. Lymphatic drainage, particularly via diaphragmatic stomata, clears CSF at rates of 1–1.5 mL/min to mediastinal lymphatics, ultimately reaching the right internal jugular and subclavian veins [9]. While VP shunts remain the mainstay of treatment, they are associated with complications including infection, mechanical obstruction, and abdominal events such as distal catheter disconnection, migration, inguinal hernia, intestinal obstruction or perforation, and CSF malabsorption–related abdominal pseudocysts and non-hepatic ascites [10, 11]. Abdominal pseudocysts are reported in 0.25% to 10.81% of VP shunt revision surgeries, with this wide range reflecting substantial heterogeneity in the literature [10, 12]. Abdominal pseudocysts are the predominant form of peritoneal malabsorption, characterized by localized, encapsulated CSF collections with a fibrous wall. Non-hepatic ascites is an extremely rare presentation in which diffuse free CSF accumulates in the peritoneal cavity without encapsulation. Although both entities share the same final pathway of impaired peritoneal absorption, non-hepatic ascites has been proposed as a distinct pathological entity. Its diagnosis is challenging, requiring abdominal distention, free fluid on imaging, and CSF-like aspirate biochemistry after exclusion of hepatic, cardiac, renal, and malignant causes. The prevailing hypothesis suggests that CSF production may exceed peritoneal absorptive capacity, thereby necessitating alternative sites for distal catheter placement, such as a ventriculoatrial (VA) shunt [13]. Severe spinal deformities (scoliosis or kyphosis) could mechanically reduce intra-abdominal volume and chronically elevate intra-abdominal pressure [14], thereby potentially decreasing the effective peritoneal surface area available for CSF absorption and possibly promoting pseudocyst formation through anatomical displacement of abdominal contents. Similarly, any previous surgery that breaches the peritoneal cavity – even seemingly minor procedures such as PEG placement, appendectomy, or inguinal hernia repair – may initiate a chronic inflammatory cascade that could lead to adhesions, fibrosis, and permanent impairment of peritoneal absorptive capacity.

This study investigates the prevalence of peritoneal CSF malabsorption, analyses potential risk factors for its occurrence, and outlines available strategies for its management.

## Methods

### Study population

Following approval from the institutional ethics committee, we conducted a retrospective review of all pediatric patients (≤ 18 years) who underwent VP shunt revision surgery at Akdeniz University Hospital between January 2019 and December 2023. Patients with two or more simultaneously functioning cranial shunts in which at least one was non-ventricular (cystoperitoneal or subdural-peritoneal shunt) were excluded for two reasons: 1- Non-ventricular shunts drain spaces (subdural hygroma, arachnoid cyst, porencephalic cyst) with unpredictable and highly variable fluid production rates compared with the relatively stable ventricular CSF production. 2- Fluid from non-ventricular spaces, particularly arachnoid cysts, often has significantly higher and more variable protein concentrations than ventricular CSF, which may independently impair peritoneal absorption and confound assessment of true peritoneal malabsorption capacity [15]. This patient selection strategy was employed to ensure a homogeneous cohort primarily defined by complications related to ventriculoperitoneal shunting, thus minimizing confounding variables arising from complex fluid dynamics in other cerebrospinal fluid pathways.

To avoid overestimating the frequency of peritoneal malabsorption in patients with multiple failed revisions during a single admission, all revision procedures performed within the same hospitalization—up to the point of definitive management with same etiology—were counted as a single event. The study cohort consisted of 81 unique pediatric patients who underwent a total of 113 VP shunt revision episodes between 2019 and 2023. Prevalence of peritoneal malabsorption was calculated using the 113 revision episodes as the denominator (11/113 = 9.7%). For risk-factor analysis, we used the 81 unique patients (each patient counted once). Within these 81 patients, 10 patients (11 revision episodes) developed peritoneal malabsorption and constitute the peritoneal malabsorption subgroup.

### Variables and definitions

Collected variables included patient age, sex, cranial catheter site (frontal or occipital), and hydrocephalus type. Congenital hydrocephalus was defined as cases without an identifiable secondary cause documented in the medical record. Acquired hydrocephalus was defined as hydrocephalus secondary to a documented event (e.g., intraventricular hemorrhage, infection, tumor, trauma) and was further subclassified as oncologic or non-oncologic, as oncologic patients may have higher shunt failure rates and potentially increased risk of peritoneal malabsorption due to factors such as chemotherapy, radiotherapy, altered CSF composition or tumor seeding [16].

Additional variables included history of multiple previous revision surgeries (≥ 2 revisions before the index episode), presence and severity of axial deformity, history of surgically treated meningomyelocele, and history of any abdominal surgery other than shunt-related procedures (e.g., PEG placement, laparotomy, appendectomy, inguinal hernia repair). Axial deformity severity was graded using Cobb angles: scoliosis — mild 10–20°, moderate 20–40°, severe > 40° [17]; kyphosis — mild 45–50°, moderate 50–75°, severe > 75° [18]. Axial deformity was included as a variable because severe kyphoscoliosis could potentially decrease abdominal cavity volume and elevate intra-abdominal pressure [14], which in turn may impair CSF absorption.

**Key operational definitions:**

#### VP shunt revision surgery

VP shunt revision surgery was defined as any operative intervention involving the shunt system, including partial or complete replacement of components, removal of the system, conversion to an alternative distal drainage site, externalization, or exploration. This definition also encompassed procedures performed for suspected shunt failure even when intraoperative findings were normal.

#### VP shunt distal dysfunction due to peritoneal malabsorption episode

surgically treated abdominal distention caused by CSF accumulation due to impaired peritoneal absorption, irrespective of whether it was accompanied by signs of acute hydrocephalus. Diagnosed by the combination of clinical abdominal distention (with/without) shunt malfunction signs, radiological findings (pseudocyst or diffuse ascites), and CSF-like aspirate biochemistry.

#### Abdominal pseudocyst

localized, encapsulated CSF collection with a fibrous wall (no epithelial lining) containing the distal catheter tip on imaging.

#### Non-hepatic Ascites

diffuse, free intra-abdominal CSF accumulation without encapsulation, with exclusion of other causes through normal liver function tests, negative cytology, normal cardiac and renal function, and absence of malignancy.

#### Revision episode

a single surgical revision or a cluster of revisions performed during the same hospitalization for the same etiology. When a new, different etiology emerged during the same admission (e.g., initial revision for cranial catheter occlusion followed by revision for cranial catheter malposition), each was counted as a separate episode.

#### Multiple revisions

defined as two or more revision episodes occurring in the same patient before the index revision episode, regardless of etiology or timing between episodes. This variable was recorded to assess whether repeated shunt interventions increase the risk of peritoneal malabsorption, potentially through cumulative peritoneal trauma or inflammation.

#### Negative outcome (malabsorption subgroup only)

requirement of ≥ 2 revision surgeries during the index admission or permanent abandonment of the peritoneal cavity with conversion to ventriculoatrial shunt.

Patients in the malabsorption group were subclassified into pseudocyst or non-hepatic ascites subgroups based on the above radiological and biochemical criteria. All malabsorption cases were followed for a minimum of 9 months with monthly abdominal ultrasonography and six-monthly cranial CT.

Statistical analysis was performed in two stages using IBM SPSS Statistics version 23. In the first stage, prevalence of peritoneal malabsorption was calculated at the revision-episode level (*n* = 113 episodes). In the second stage, risk-factor analysis was performed at the patient level (*n* = 81 unique patients) to identify factors associated with the development of peritoneal malabsorption, followed by analysis of the peritoneal malabsorption subgroup (10 patients) to identify predictors of management outcome (positive vs. negative).

## Results

### Characteristics of the overall cohort and prevalence of peritoneal malabsorption (episode-level analysis, *n* = 113)

Between January 2019 and December 2023, 81 unique pediatric patients (41 males, 40 females; median age 7 years, range 0–18 years) underwent a total of 113 VP shunt revision episodes at our institution. Peritoneal malabsorption was the indication for revision in 10 patients (accounting for 11 revision episodes), corresponding to a prevalence of 9.7% when calculated at the episode level (11/113) (Table 1).

**Table 1:** Demographic and clinical characteristics of the cohort

Variable	Value
No. of patients	81
Median age (year)	7.0 years (range 0–18)
Female/Male	(40/81) (49.4%) / (41/81) (50.6%)
Total No. of revision episodes	113
Patients with 1 revision episode	57
Patients with 2 revision episodes	17
Patients with 3 revision episodes	6
Patients with 4 revision episodes	1
Average No. of revision episodes per patient	1.39
Congenital hydrocephalus (episodes)	68/113 (60.2%)
Acquired hydrocephalus (episodes)	45/113 (39.8%)
Cranial catheter site – frontal (episodes)	45/113 (39.8%)
Cranial catheter site – occipital (episodes)	68/113 (60.2%)

The most common causes of revision are presented in Table 2. Obstruction/occlusion (confirmed intraoperatively) was the leading cause (35 episodes, 31%), subdivided into cranial catheter (*n* = 17), valve (*n* = 7), and peritoneal catheter (*n* = 11). Distal catheter shortening due to somatic growth accounted for 20 episodes (17.7%).

**Table 2:** Causes of VP shunt revision (n = 113 episodes) *

Causes of shunt revision	Frequency	Percent (%)
Obstruction/occlusion	35	31
Shortening of peritoneal catheter**	20	17.7
Peritoneal malabsorption	**11**	**9.7**
Infection	9	8
Disconnection***	9	8
Shunt exploration-no no dysfunction***	8	7.1
Valve replacement (programmable)	5	4.4
Others****	16	14.2

*All categories are defined as mutually exclusive; obstruction/occlusion was limited to intraoperatively confirmed blockage, while shortening, disconnection, migration, and malposition were classified separately and not counted within obstruction.

**Shortening: Radiographically confirmed distal catheter too short due to growth, not reaching peritoneum

***Disconnection: Radiographic/intraoperative separation of components

** Shunt exploration – no dysfunction found = procedures performed for clinically/radiologically suspected shunt failure even when intraoperative findings were completely normal

**** Others include skin maceration (8), cranial catheter malposition (4), peritoneal catheter migration (1), shunt removal due to unnecessity (1), neck movement limitation due to shunt trace calcification (1), and shunt damage during central venous catheter placement (1)

### Risk factors for peritoneal malabsorption (patient-level analysis, *n* = 81)

Univariate logistic regression identified three statistically significant risk factors (Table 3):

- History of multiple previous shunt revisions (≥ 2 before the index episode): OR 8.02 (95% CI 1.86–34.63, *p* = 0.002).
- Prior non-shunt-related abdominal surgery that breached the peritoneal cavity: OR 11.81 (95% CI 2.73–51.04, *p* = 0.001).
- Presence of axial deformity (any severity): OR 4.07 (95% CI 1.03–16.03, *p* = 0.034), with a 2.77-fold increase per severity grade (95% CI 1.12–6.85, *p* = 0.027).

**Table 3:** Univariate risk factors for peritoneal malabsorption (patient-level analysis, n = 81)

Variable*	Category	Non-malabsorption (*n* = 71) n (%)	Malabsorption (*n* = 10) n (%)	OR (95% CI)	P Value
Age (years)	Continuous (Mean ± SD)	7.77 ± 5.39	7.60 ± 4.53	1.01 (0.93–1.09)	0.922
Sex	Male vs. Female	Male 35 (49.3%)	Male 6 (60.0%)	0.648 (0.168–2.495)	0.526
Cranial catheter site	Frontal vs. Occipital	Frontal 30 (42.3%)	Frontal 4 (40.0%)	1.098 (0.285–4.234)	0.892
Hydrocephalus etiology	Congenital vs. Acquired	Congenital 42 (59.2%)	Congenital 6 (60.0%)	0.966 (0.250–3.727)	0.959
History of ≥ 2 previous revisions	Yes vs. No	Yes 16 (22.5%)	Yes 7 (70.0%)	8.021 (1.858–34.627)	0.002
Axial deformity (any severity)	Yes vs. No	Yes 14 (19.7%)	Yes 5 (50.0%)	4.071 (1.034–16.031)	0.034
Axial deformity severity	Per grade increase	—	—	2.77*** (1.12–6.85)	0.027**
Cranial-abdominal infection	Yes vs. No	Yes 8 (11.3%)	Yes 3 (30.0%)	3.375 (0.724–15.737)	0.105
History of operated meningomyelocele	Yes vs. No	Yes 12 (16.9%)	Yes 1 (10.0%)	0.55 (0.06–4.72)	0.578
Prior peritoneal-breaching abdominal surgery	Yes vs. No	Yes 8 (11.3%)	Yes n =(6) (60.0%)	11.813 (2.73–51.04)	**0.001**

*Age and axial deformity severity were analysed by binary logistic regression; all other variables by χ² test or Fisher’s exact test. Significant p-values (*p* < 0.05) are bolded

**Binary logistic regression was used to estimate odds ratios (ORs) by exponentiating the regression coefficients (Exp(B))

*** Each one-step increase in deformity severity (mild → moderate, or moderate → severe) is associated with a 2.77-fold increase in the odds of developing malabsorption

Prior non-shunt-related abdominal surgery that breached the peritoneal cavity was present in 6 of 10 patients (60%) who developed peritoneal malabsorption versus 8 of 71 patients (11.3%) without malabsorption. The distribution of specific procedures was as follows:

#### PEG placement

4 patients in the malabsorption subgroup vs. 1 patient in the non-malabsorption subgroup (all endoscopic).

#### Appendectomy

1 patient in the malabsorption subgroup (open) vs. 3 in the non-malabsorption subgroup (1 open, 2 endoscopic).

#### Inguinal hernia repair

1 patient in the malabsorption subgroup vs. 4 patients in the non-malabsorption subgroup ( all endoscopic).

All procedures had been performed at any time point before the index revision surgery (range 3 months to 8 years).

History of necrotizing enterocolitis (NEC) was observed in 3 patients (all in the non-malabsorption subgroup). These episodes were managed medically in the neonatal period, preceding shunt placement by years, and showed no association with the development of peritoneal malabsorption.

To address concern regarding the potential influence of our patient-level approach on risk-factor estimates, a sensitivity analysis was performed considering each revision episode as an independent event (*n* = 113 episodes). The three identified risk factors remained statistically significant, with similar or stronger associations: prior abdominal surgery OR ≈ 14.5 (95% CI 3.65–57.47, *p* < 0.001), axial deformity OR ≈ 4.93 (95% CI 1.36–17.86, *p* = 0.009), history of ≥ 2 previous revisions OR ≈ 27.8 (95% CI 5.26–146.28, *p* < 0.001) These concordant results confirm the robustness of the findings.

### Management and outcomes in the peritoneal malabsorption subgroup (10 patients)

The mean hospital stay duration was 8.5 days for the overall cohort and 40.6 days (range 14–119 days) for the malabsorption subgroup (calculated at the revision-episode level).

Management followed the algorithm shown in Fig. 2.

- Externalization with 2–4 weeks of temporary drainage was performed in 6 episodes (massive pseudocysts or infection concern), with successful peritoneal re-implantation as a first single surgery in 3 episodes.
- Mini-laparotomy with adhesiolysis and immediate re-implantation was attempted in 4 episodes, successful as a first single surgery in 1 episode.
- Subdiaphragmatic placement (same session) was successful as a first single surgery in 1 episode.

Successful peritoneal salvage was achieved in 7 of 10 patients. Five episodes were managed with a single surgical procedure, while 6 episodes required multiple interventions, with 3 ultimately needing ventriculoatrial shunt conversion (Table 4). Negative outcome (≥ 2 revision operations during the index admission or permanent abandonment of the peritoneum) occurred in 6 episodes. No variable reached statistical significance for predicting negative outcome.

**Table 4:** Characteristics and management outcomes of the 11 peritoneal malabsorption revision episodes (10 patients)

Episode No.	sex	Cranial catheter site	Malabsorption type	Hydrocephalus etiology	History of ≥ 2 previous revisions	Axial deformity (severity)	History of cranial-abdominal infection	Prior peritoneal-breaching abdominal surgery	No. of revision procedures during index admission	Definitive management	Follow-up duration (months)
1	F	Occipital	Non-hepatic ascites	Congenital	No	Yes (severe)	No	No	3	VA shunt	12
2	M	Occipital	Pseudocyst	Congenital	No	Yes (severe)	No	No	1	Subdiaphragmatic reimplantation	25
3	M	Frontal	Pseudocyst	Congenital	Yes	Yes (severe)	No	Yes	3	VA shunt	12
4	M	Occipital	Pseudocyst	Congenital	yes	No	Yes	yes	1	adhesiolysis with immediate reimplantation	13
5	M	Occipital	Pseudocyst	Congenital	Yes	No	No	Yes	2	Subdiaphragmatic reimplantation	13
6	M	Frontal	Pseudocyst	Congenital	No	No	No	Yes	1	Replacement to peritoneum after 2–4 weeks of externalization	12
7	F	Frontal	Pseudocyst	Acquired - oncologic	Yes	No	No	No	1	Replacement to peritoneum after 2–4 weeks of externalization	9
8	F	Occipital	Pseudocyst	Acquired – non oncologic	Yes	Yes (moderate)	Yes	Yes	4	Subdiaphragmatic reimplantation	26
9*	F	Occipital	Pseudocyst	Acquired – non oncologic	Yes	Yes (moderate)	Yes	Yes	2	adhesiolysis with immediate reimplantation	25
10	M	Occipital	Pseudocyst	Acquired - oncologic	Yes	Yes (mild)	No	No	1	Replacement to peritoneum after 2–4 weeks of externalization	16
11	F	Frontal	Pseudocyst	Acquired – non oncologic	Yes	No	Yes	Yes	4	VA shunt	16

* Episodes 8 and 9 occurred in the same patient, separated by 26 months

## Discussion

**Fig. 1:**
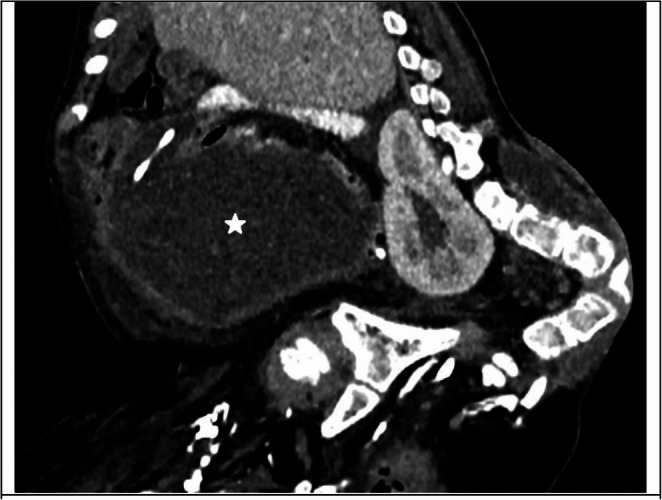
An abdominal CT scan of a 9-year-old male patient (patient no. 3) demonstrates an abdominal pseudocyst (asterisk) containing the distal catheter. The patient has sacral agenesis and severe lumbar kyphosis, with a history of both prior abdominal surgery and multiple revision surgeries. In this case, the peritoneal cavity could not be salvaged, and the patient ultimately underwent conversion to a VA shunt

Peritoneal malabsorption remains one of the most challenging complications of ventriculoperitoneal shunting in children. The pathophysiology of abdominal pseudocyst formation is multifactorial and likely involves a chronic inflammatory response to prior peritoneal injury (e.g., surgery), low-grade infection (even with negative cultures), or reaction to shunt material or CSF components (e.g., elevated protein) [19–21]. These processes lead to fibrous encapsulation around the distal catheter tip and localised CSF collection. In this single-center series of 113 revision episodes, peritoneal malabsorption accounted for 9.7% of episodes, a figure consistent with the wide range (0.25–10.8%) reported in the literature [10, 12]. Three factors were statistically associated with the development of peritoneal malabsorption on univariate patient-level analysis: history of multiple previous revisions, prior peritoneal-breaching abdominal surgery, and presence of axial deformity. The strongest association was observed with any previous surgery that violated the peritoneal cavity (OR 11.8, 95% CI 2.7–51.0). Remarkably, the association extended even to procedures traditionally regarded as minor (PEG placement 4 vs. 1, appendectomy 1 vs. 3, inguinal hernia repair 1 vs. 4), suggesting that any breach of the peritoneal lining – regardless of extent or timing – can initiate a chronic inflammatory cascade that reduces functional absorptive surface area through adhesions and fibrosis. Percutaneous Endoscopic Gastrostomy (PEG) insertion constitutes a temporary breach of the peritoneum with a risk of microbial contamination and subclinical gastric micro-leakage, documented by transient post-procedure pneumoperitoneum [22]. The resulting irritation of the peritoneum, whether from prior surgery (such as PEG), repeated surgical revisions, or even the chronic presence of cerebrospinal fluid components [23], may sustain a localized, low-grade inflammatory state. Such persistent peritoneal inflammation triggers injury repair and reconstruction, leading to pathological structural changes in the peritoneum, including a decrease in mesothelial cells, an increase in fibers under the mesothelium, and neovascularization [24]. These fibrotic changes around the stoma theoretically compromise the long-term integrity and normal absorptive/filtering function of the peritoneal membrane in the immediate area. A history of multiple previous revisions (≥ 2 before the index episode) was also significantly associated (OR 8.02, 95% CI 1.86–34.63). Repeated surgical interventions on the shunt system may, through cumulative peritoneal trauma, exacerbate inflammation and potentially promote the release of pro-fibrotic mediators, leading to peritoneal thickening, adhesions, and fibrosis – mechanisms analogous to those observed with prior abdominal surgery. Severe axial deformity also emerged as an independent risk factor (OR 4.07 for presence; OR 2.77 per severity grade). The underlying mechanism is unlikely to be simple catheter kinking, but rather a combination of chronically elevated intra-abdominal pressure and anatomical displacement of abdominal contents. Both phenomena could limit the effective low-pressure peritoneal surface available for CSF absorption and promote localised pseudocyst formation through omental or bowel wrapping of the catheter tip. (Fig. 1)

Non-hepatic ascites was observed in only one patient in our cohort. This entity, while sharing the same final pathway of impaired peritoneal absorption as pseudocysts, poses a significant diagnostic and management challenge. In this case, the patient presented with massive abdominal distention, shifting dullness, and diffuse anasarca on imaging. Paracentesis yielded clear CSF-like fluid, with a serum-ascites albumin gradient (SAAG) found to be (≈ 2.4 g/dL) > 1.1 g/dL. Extensive work-up—including normal liver biochemical tests, complete blood count, urinalysis, hepatic Doppler, echocardiography, and invasive hepatic venous pressure measurements—ruled out portal hypertension, hepatic disease, cardiac, renal, or malignant causes. Despite a normally functioning shunt on study, the peritoneum could not be salvaged despite multiple attempts. No conclusions can be drawn about its specific mechanism or optimal management from this single case.

**Fig. 2:**
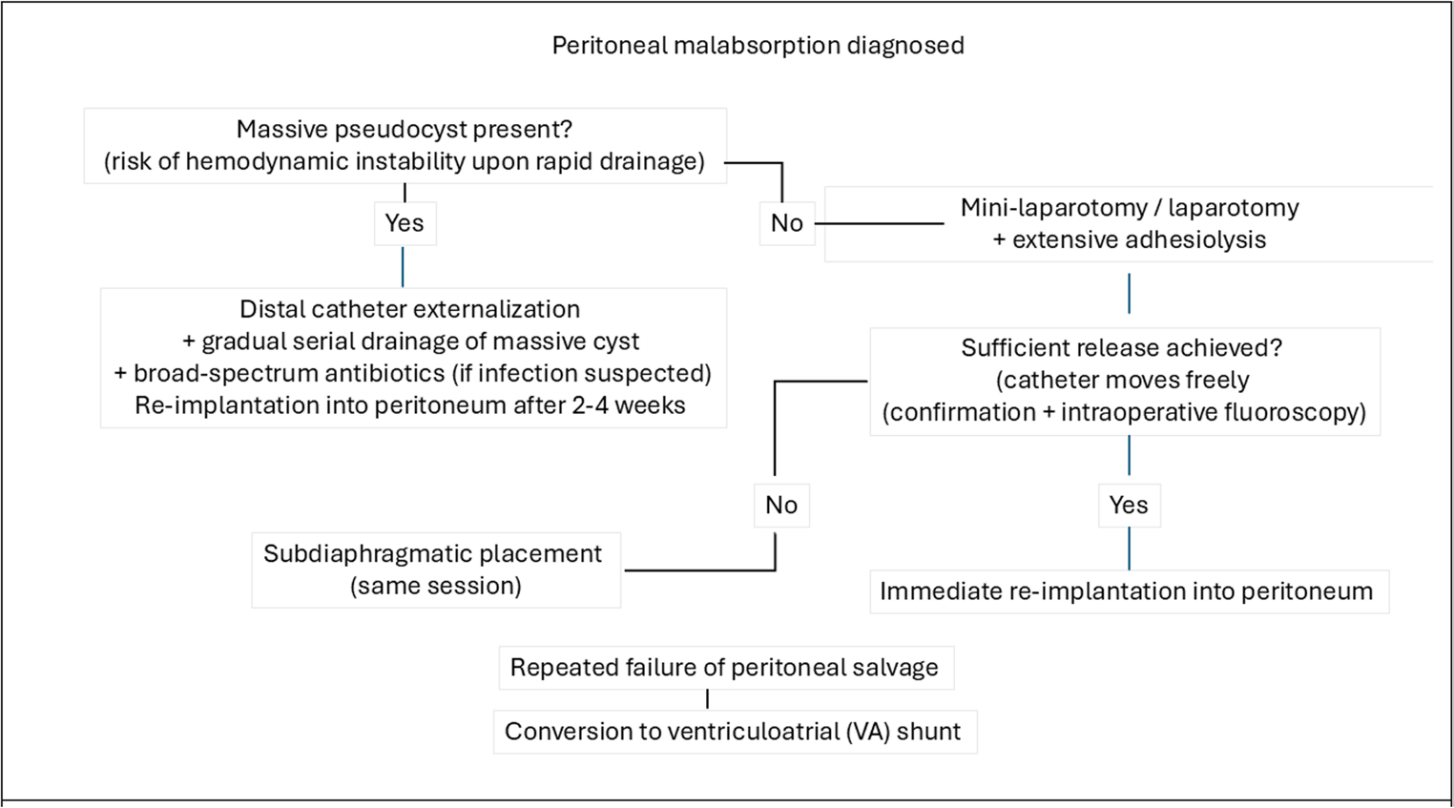
Institutional management algorithm for peritoneal malabsorption in pediatric VP-shunted patients. Massive pseudocyst” = localized CSF collection of such volume that rapid single-session drainage could theoretically cause hemodynamic instability due to sudden drop in intra-abdominal pressure (although this complication has not been reported in the shunt literature, we adopt a cautious approach). Decision between immediate re-implantation and subdiaphragmatic placement is based on intraoperative assessment of adhesiolysis adequacy

**Fig. 3:**
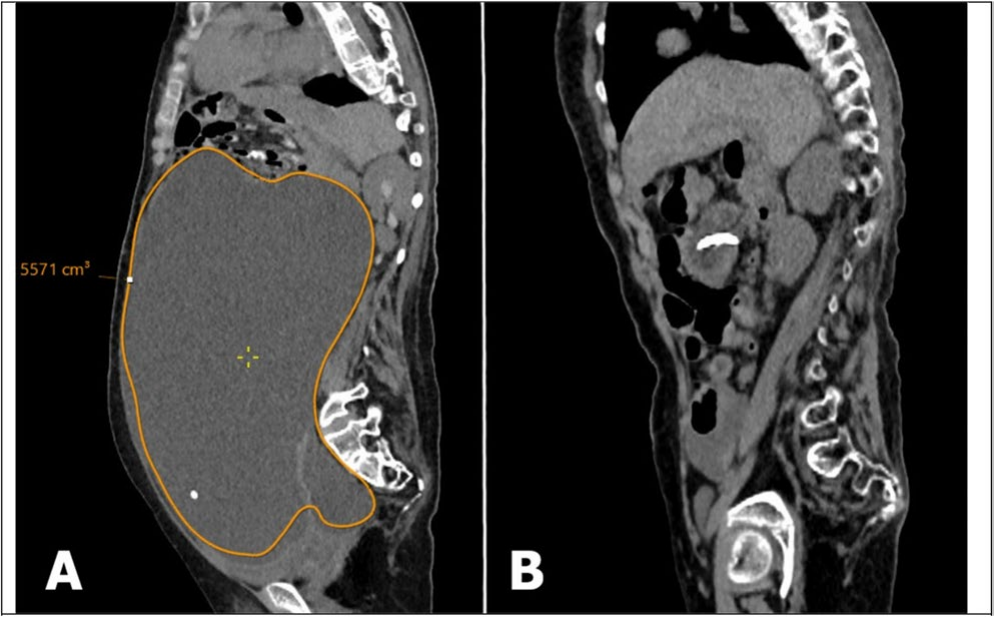
Radiological illustration of massive abdominal pseudocyst and resolution. **A**. pre-operative abdomen sagittal CT scan demonstrated a massive abdominal pseudocyst with an estimated volume of 5.5 L. The distal (VP) shunt catheter tip was visible inside the collection. **B**- post-operative abdomen sagittal CT scan scan performed following cautious, serial drainage management and definitive revision demonstrated near-complete resolution of the massive pseudocyst. However, the pseudocyst recurred within 10 days during the same hospital admission, necessitating multiple adhesiolysis procedures before definitive management was achieved through subdiaphragmatic reimplantation (Episode 8, Table 4)

Management aimed to preserve the peritoneal cavity whenever possible (Fig. 2). The decision between immediate re-implantation and prolonged externalization hinged on two features:

1. Presence of a massive pseudocyst (Fig. 3) (defined as a localised collection large enough to potentially cause hemodynamic instability upon rapid single-session drainage due to sudden drop in intra-abdominal pressure – a theoretical risk that has not been reported in the shunt literature but is managed cautiously in our practice. Although CSF accumulation does not share the same primary pathophysiology as hepatic ascites, the rapid drop in intra-abdominal pressure and resulting splanchnic venous pooling may share a similar mechanism for acute hemodynamic decompensation. This risk is estimated by comparison to the pediatric ascites literature, where a volume exceeding 200 ml/kg of dry body weight is strongly associated with circulatory dysfunction) [25].
2. Suspicion of active infection. If either of these conditions was present—massive pseudocyst or suspected infection—the VP shunt was externalized with serial drainage for 2–4 weeks, during which at least three consecutive clear CSF samples were required before distal catheter re-implantation was performed.

When neither a massive pseudocyst nor infection concern was present, adhesiolysis was performed as the next step in the management strategy. If adequate release was achieved—allowing free catheter movement throughout the peritoneal cavity (confirmed visually and by intraoperative fluoroscopy)—immediate re-implantation was undertaken in the same session. This approach is consistent with the goal of preserving the peritoneal cavity whenever technically feasible, thereby accounting for cases managed in a single operative procedure. Ultimately, peritoneal salvage was successful in 7 of 10 patients (70%). Conversion to a ventriculoatrial shunt was required in the remaining three. No preoperative or intraoperative variable reliably predicted the need for multiple procedures or eventual abandonment of the peritoneum.

### Limitations and future directions

This study has several important limitations. It is a retrospective, single-center series with a small number of malabsorption events (10 patients / 11 episodes), resulting in wide confidence intervals and limited statistical power. The risk-factor assessment was based on univariate analysis only, without multivariable adjustment or correction for multiple testing. Management was not protocolized and reflects institutional practice rather than a standardised approach. A notable limitation is the use of the individual patient as the unit of analysis in the primary risk-factor assessment, with recurrent revisions from the same patient counted only once. This is a common practice to avoid overestimation and clustering effects. However, our sensitivity analysis, which utilized the revision episode as the unit of analysis (*n* = 113 episodes), confirmed that the significant risk factors identified (prior abdominal surgery, axial deformity, and history of multiple previous revisions) remained statistically significant, with similar or stronger associations. This indicates that the main findings of this study are robust to the choice of analytical unit. Consequently, the reported associations must be regarded as exploratory and hypothesis-generating rather than definitive. Larger, prospective, multicenter studies are needed to confirm these findings and to develop evidence-based management algorithms.

## Conclusion

In this small single-center retrospective series, peritoneal malabsorption accounted for approximately 9.7% of VP shunt revision episodes in pediatric patients. Prior peritoneal-breaching abdominal surgery, multiple previous revisions and axial deformity were statistically associated with its development, possibly through inflammatory and mechanical mechanisms that reduce effective absorptive surface area. Management remains challenging and individualised, with peritoneal salvage achievable in the majority of cases using a stepwise approach that prioritises cautious handling of massive pseudocysts and adequate adhesiolysis. Affected patients experienced markedly prolonged hospitalization - mean 40.6 days (range 14–119 days) per malabsorption revision episode compared with 8.5 days for the overall cohort, underscoring the substantial clinical burden of this complication. These preliminary observations highlight the multifactorial nature of peritoneal malabsorption and underscore the importance of preoperative assessment of surgical history and spinal deformity. Larger, prospective studies are essential to validate these associations, elucidate underlying mechanisms, and establish standardised therapeutic strategies.

## Data Availability

A dataset was generated from patient records at Akdeniz University Hospital (2019–2023). Data are not publicly available due to patient confidentiality but may be accessed upon reasonable request, subject to institutional approval.
